# Author Correction: Isogambogenic acid induces apoptosis-independent autophagic cell death in human non-small-cell lung carcinoma cells

**DOI:** 10.1038/s41598-020-62992-y

**Published:** 2020-04-20

**Authors:** Jianhong Yang, Yongzhao Zhou, Xia Cheng, Yi Fan, Shichao He, Shucai Li, Haoyu Ye, Caifeng Xie, Wenshuang Wu, Chunyan Li, Heying Pei, Luyuan Li, Zhe Wei, Aihua Peng, Yuquan Wei, Weimin Li, Lijuan Chen

**Affiliations:** 1 State Key Laboratory of Biotherapy/Collaborative Innovation Center of Biotherapy and Cancer Center, West China Hospital of Sichuan University, Chengdu, China; 2 Department of Respiratory Medicine, West China Hospital of Sichuan University, Chengdu, China; 3 State Key Laboratory of Medicinal Chemical Biology and Nankai University College of Pharmacy, Tianjin, China

Correction to: *Scientific Reports* 10.1038/srep07697, published online 09 January 2015

This Article contains errors in Figure 2B and Supplementary Figure S1A. In Figure 2B, the image for Iso-GNA(2.5μM) is a duplication of the Control. The correct Figure 2B appears below as Figure 1.

**Figure 1:**
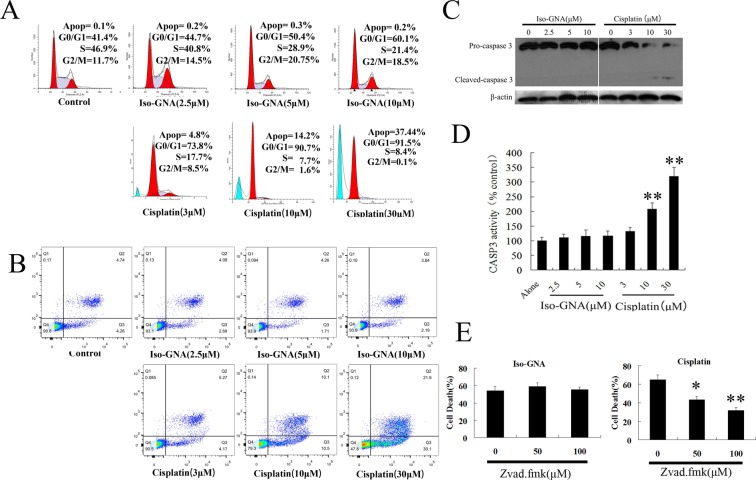
.

In Supplementary Figure S1A, the image for Iso-GNA(5μM) is a duplication of Iso-GNA(2.5μM). The correct Supplementary Figure S1A appears below as Figure 2.

**Figure 2:**
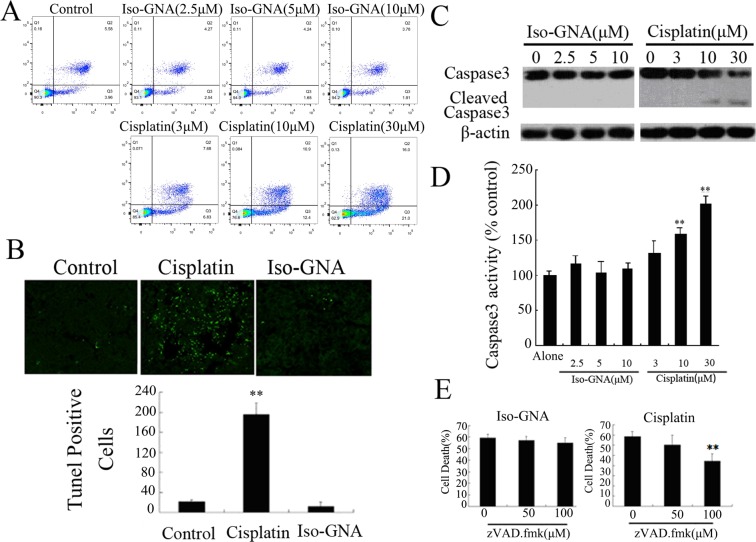
.

To produce the corrected figures, the authors re-processed the data resulting in the number shift.

